# Inferring the Demographic History and Rate of Adaptive Substitution in *Drosophila*

**DOI:** 10.1371/journal.pgen.0020166

**Published:** 2006-10-13

**Authors:** Haipeng Li, Wolfgang Stephan

**Affiliations:** Section of Evolutionary Biology, Department of Biology II, University of Munich, Planegg-Martinsried, Germany; University of Chicago, United States of America

## Abstract

An important goal of population genetics is to determine the forces that have shaped the pattern of genetic variation in natural populations. We developed a maximum likelihood method that allows us to infer demographic changes and detect recent positive selection (selective sweeps) in populations of varying size from DNA polymorphism data. Applying this approach to single nucleotide polymorphism data at more than 250 noncoding loci on the X chromosome of Drosophila melanogaster from an (ancestral) African population and a (derived) European, we found that the African population expanded about 60,000 y ago and that the European population split off from the African lineage about 15,800 y ago, thereby suffering a severe population size bottleneck. We estimated that about 160 beneficial mutations (with selection coefficients *s* between 0.05% and 0.5%) were fixed in the euchromatic portion of the X in the African population since population size expansion, and about 60 mutations (with *s* around 0.5%) in the diverging European lineage.

## Synopsis

The authors provide evidence for the recent action of positive selection in the fruit fly Drosophila melanogaster. They describe a new statistical method to detect footprints of selection in the genome of this species and apply it to a large set of DNA polymorphism data from the X chromosome. They find that numerous selective events occurred in the past 60,000 y, while D. melanogaster expanded its ancestral range in Africa and subsequently colonized temperate zones in the rest of the world after the last ice age. The findings of this paper are important as they indicate where in the genome the genes are located that have been involved in the adaptation of D. melanogaster to environmental changes in the recent past. The approach developed here for the model species D. melanogaster can be readily extended to study adaptation in humans and other species.

## Introduction

A long-standing interest in evolutionary biology has been to estimate the rate of adaptive substitution. Adaptive events can be inferred from interspecific data by comparing nonsynonymous and synonymous substitution rates [1]. A second approach has been to use a combination of both interspecific and intraspecific data in employing the McDonald-Kreitman method [2]. It has been found that positive selection could play a role in the human-chimpanzee lineages [3] and that as much as 45% of all amino-acid substitutions have been fixed by natural selection in *Drosophila* [4]. However, the methods that include interspecific data (in particular, the McDonald-Kreitman test) may be sensitive to fairly small fluctuations in effective population size and other demographic changes [5]. An alternative is to use only data on intraspecific variation and to explicitly model the effects of demographic changes and positive selection [6–8].

Footprints of *very recent* positive selection can be detected by identifying selective sweeps in the genome (in particular, valleys of reduced polymorphism). In the last few years, several methods have been proposed to detect selective sweeps [9–13]. To distinguish signatures of sweeps from those of demography and estimate the rate of adaptive substitutions, we use here a modification of the approach of Li and Stephan [12].

Reduced polymorphism due to hitchhiking will be restored after about 0.1*N_e_* generations [9,14]. This feature enables us to detect very recent hitchhiking events and to reveal the relationship between adaptation and habitat change in a species that invaded new territory. For these purposes, the cosmopolitan species D. melanogaster serves as an appropriate model since this species, originally from Africa, expanded its population size worldwide very recently [15,16]. We analyzed DNA polymorphism at more than 250 noncoding loci on the X chromosome from two D. melanogaster populations: the Netherlands and east Africa [16–18]. The homologous sequences of D. simulans are used as outgroup data to infer the ancestral status of a polymorphic site and to estimate divergence between D. melanogaster and D. simulans.

## Results/Discussion

### Inferring Demography: General Approach

Demographic change affects the genome-wide polymorphism pattern in a species or population. Thus, we used the whole dataset to infer demographic processes in the two populations. For the African population, the dataset is given in terms of the mutation frequency spectrum (MFS), where the MFS is the distribution describing the relative abundance of derived mutations occurring *i* = 1, 2, …, *n* − 1 times in *n* homologous sequences.

Following Nielsen [19], the likelihood for the *k*th locus is given as 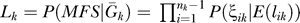 , where 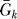 is a set of (*n_k_* − 1) expected branch lengths [12] under the demographic scenario. The branch length is scaled so that one unit represents 2*N_A_*_0_ generations, where *N_A_*_0_ is the current effective population size for the X chromosome in the African population; *n_k_* is the sample size of the *k*th locus, ξ*_ik_* is the number of derived mutations carried by *i* sampled chromosomes for the *k*th locus, and *E(l_ik_)* is the expected length of branches with *i* descendants for the *k*th locus under the demographic scenario. *P*(ξ*_ik_*|*E(l_ik_)* is given by the Poisson probability, i.e., 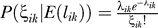 , with λ*_ik_* = *E(l_ik_)*θ*_Ak_*/2, which is the expected number of derived mutations occurring *i* times in *n_k_* sampled sequences at the *k*th locus, where θ*_Ak_* = 4*N_A_*_0_ξ*_ik_*, and μ*_k_* is the mutation rate of the *k*th locus. Since loci are independent given the expected branch lengths, the likelihood for all loci is 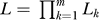 , where *m* is the number of loci.

**Figure 1:**
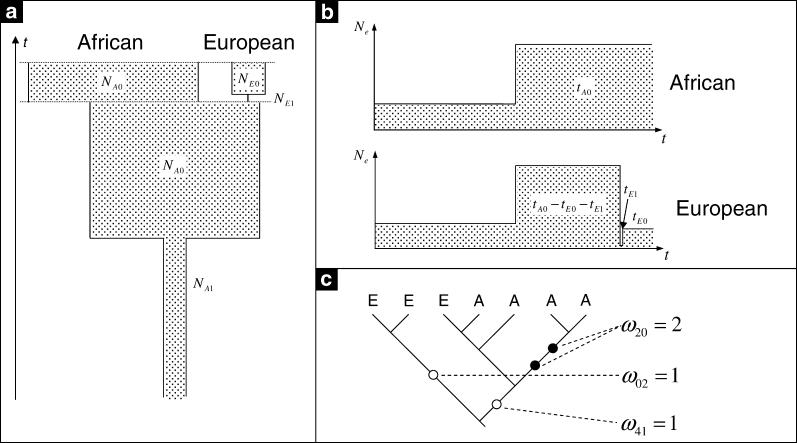
Demographic Models of the African and European Populations (A) The demographic histories are plotted together. (B) The demographic histories are plotted for both populations separately. (C) The joint MFS for an example genealogy where the sample size of European lines (indicated by E) is 3, and that of African lines (A) is 4. ω*_ij_* is the number of mutations carried by *i* chromosomes of the African sample and by *j* chromosomes of the European sample.

To infer the demographic change in the derived European population, we used the *joint* MFS [20] (Figure 1). If the sample sizes of the African and European populations are *n_A_* and *n_E_* (*n_A_* ≥ 0 and *n_E_* ≥ 0), respectively, the joint MFS for one locus is

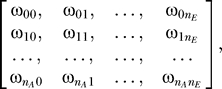
where ω*_ij_* is the number of derived mutations carried by *i* sampled chromosomes in the sample from the African population and by *j* sampled chromosomes in the sample from the European population. The values of ω_00_ ω*_n_A___n_E__* and (denoting the numbers of mutations that are not present and fixed in the sample, respectively) are not considered in the analysis.

Finally, we assume that the out-of-Africa migration does not affect the genetic polymorphism in the African population (Figure 1). This is reasonable because the size of the founder population is likely to be very small compared to the size of the ancestral African population. Thus, we estimated the demographic scenario of the European population conditional on the estimated demographic scenario of the African population. Under this assumption, the likelihood for the joint MFS is calculated in a similar way as described above (see Materials and Methods).

### Demographic History of the African Population

**Figure 2:**
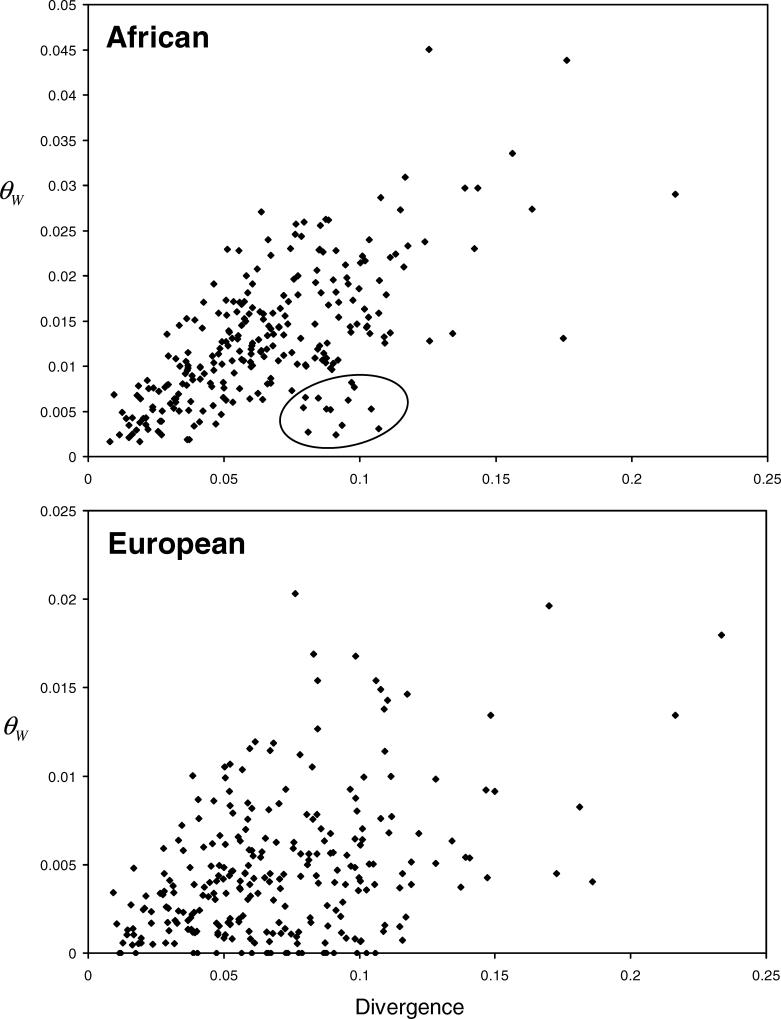
Watterson's θ*_W_* versus Divergence between D. melanogaster and D. simulans Pearson's *r* = 0.65, *p* < 0.01 in the African sample; Pearson's *r* = 0.40, *p* < 0.01 in the European sample. In the African sample, 13 loci are circled because of their high divergence and low θ*_W_*.

Before entering the analysis, it is crucial to examine whether the mutation rate among the noncoding loci is homogeneous. We found that the level of genetic polymorphism of a locus (measured by Watterson's θ*_W_*) is significantly positively correlated with divergence between D. melanogaster and D. simulans (Figure 2). Based on the Poisson distribution, we compared the mutation rate μ*_k_* of each locus *k* (estimated from divergence) with the average mutation rate 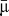 of loci over the whole X chromosome (i.e., the average of mutation rates across loci weighted by sequence length). The null hypothesis is 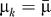 , which is tested using Monte-Carlo simulations. The estimated mutation rate of 62 of the 266 loci (23.3%) is significantly lower than the average (at 1% significance level, one-tailed test), while that of 51 of the 266 loci (19.2%) is significantly higher. This suggests that the mutation rate among loci is not homogeneous. Therefore, we used two models in the following analysis: (i) a constant mutation rate model in which the mutation rate of each locus is 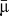 and (ii) a varying mutation rate model in which the mutation rate of locus *k* is μ*_k_*. The constant mutation rate model underestimates the variance of mutation rates among loci while the varying mutation rate model overestimates this variance (because of the sampling error of the estimated mutation rates).

**Figure 3:**
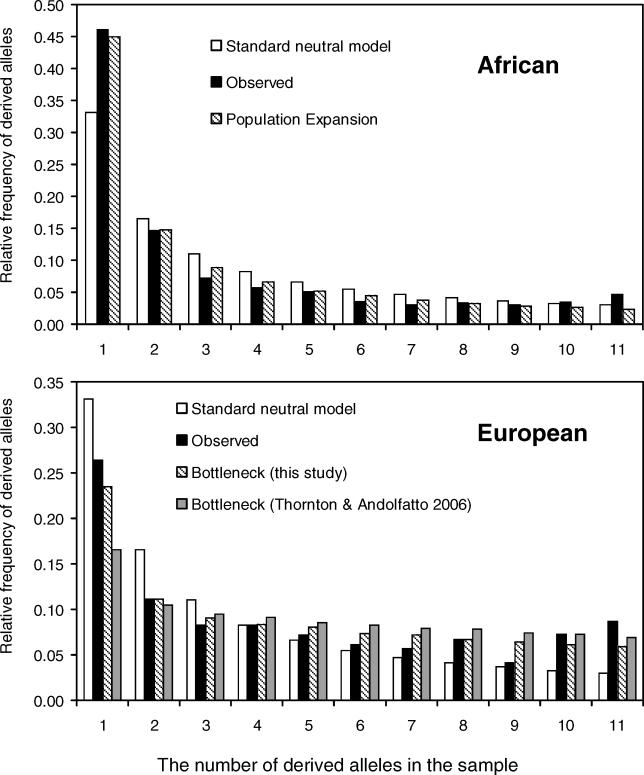
Comparisons of MFS For the European population, the expected MFSs under our estimated bottleneck scenario and a previously proposed bottleneck scenario [24] are plotted.

Compared with the standard neutral model, a genome-wide excess of rare derived mutations in the African sample is observed [16] (Figure 3). This may indicate that the African population has expanded in recent time [6] or been affected by purifying selection. To examine the first hypothesis, we compared an instantaneous population expansion model (Figure 1) with the standard neutral model. That is, the null hypothesis is N*_A_*_0_ = N*_A_*_1_ (the simple model), and the alternative hypothesis is N*_A_*_0_ ≥ N*_A_*_1_ (the complex model). The likelihood ratio test (LRT) (*p* < 0.01, *df* = 1) suggests that the expansion model [max log*(L)* = −4,181.5] explains the features of the polymorphism data in the African sample significantly better than the standard neutral model [max log*(L)* = −4,324.0].

In the expansion model, the estimated 
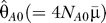
 is 0.0499. 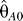 is larger than the observed Watterson's θ*_W_* (0.0126) because the population is not in mutation-drift equilibrium. Since the D. melanogaster lineage split from D. simulans approximately 2.3 million years ago [21] and the averaged divergence over loci is 0.0667, 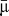 is 1.450 × 10^−9^ per site per generation (assuming ten generations per year). Then 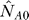 is 8.603 × 10^6^, and the estimated time to the expansion in the past 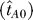 is 60,000 y with a 95% confidence interval (CI) of 26 to 95 ky. The strength of the expansion, measured by the ratio of the current size to the size before the expansion, is 5.0 (3.5 – 8.5).

An assumption of the above analysis is that all single nucleotide polymorphisms evolve neutrally. However, it may well be that part of the excess of rare mutations seen in the African population is due to purifying selection. For this reason, following Fu [22] and Smith and Eyre-Walker [4], we repeated the analyses disregarding the singletons (i.e.*,* the mutations carried by a single chromosome in the sample). In this case, the power to reject the standard neutral model is lower but the test is still significant (*p* < 0.05). The strength of the expansion (6.5) is slightly higher than that estimated above, while the time of expansion in the African population was almost unchanged (60,000 y versus 56,000 y). This may suggest that population size expansion has a larger effect on variation than purifying selection.

To gauge the error in estimating the likelihood function, we calculated the variance of log-likelihood (given the estimated parameters) for the African population. It is 0.0087, a rather small value relative to the max log(*L*) value (obtained above).

### Demographic History of the European Population

After combining the European and African datasets, the demographic history of the European population is inferred using the joint MFS (Figure 1) and assuming that all single nucleotide polymorphisms evolve neutrally. The joint MFS contains more information than the MFS of the European sample alone because the former also includes information on population divergence. The maximum likelihood estimate of 
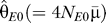
 is 0.0062, and the estimated current effective population size for the X chromosome 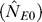 of the European population is 1.075 × 10^6^. The African and European populations diverged approximately 15,800 y ago (12 to 19 kya). The effective size of the founder population was only 2,200 (540 to 10,800), and the duration of the bottleneck about 340 y (20 to 1,000 y).

In the following, we discuss the results of our demographic analyses. The methods for inferring the demographic scenarios for the African and European populations go beyond our previous study [18]. First, because the constant-size model could not be rejected in the previous analysis [18], it is likely that our method has higher power to detect demographic changes than the Weiss–von Haeseler approach [23] we used previously. Second, the most important progress is that we used the joint MFS. Thus, we avoided the difficulty in inferring mutations that occurred in the European population after the two populations diverged. Third, in the bottleneck model for the derived European population, it is no longer necessary to assume equal prebottleneck and postbottleneck population sizes as previously done [18].

Our estimate of the divergence time (but not of the duration of the bottleneck and the size of the European founder population) agrees with a value recently reported [24]. In Figure 3, we compare the implications of these two bottleneck scenarios. Because the sum of the squares of the residuals for our bottleneck scenario is lower (0.003 versus 0.013), our bottleneck scenario explains the overall polymorphism pattern on the X chromosome better than the other one.

**Figure 4:**
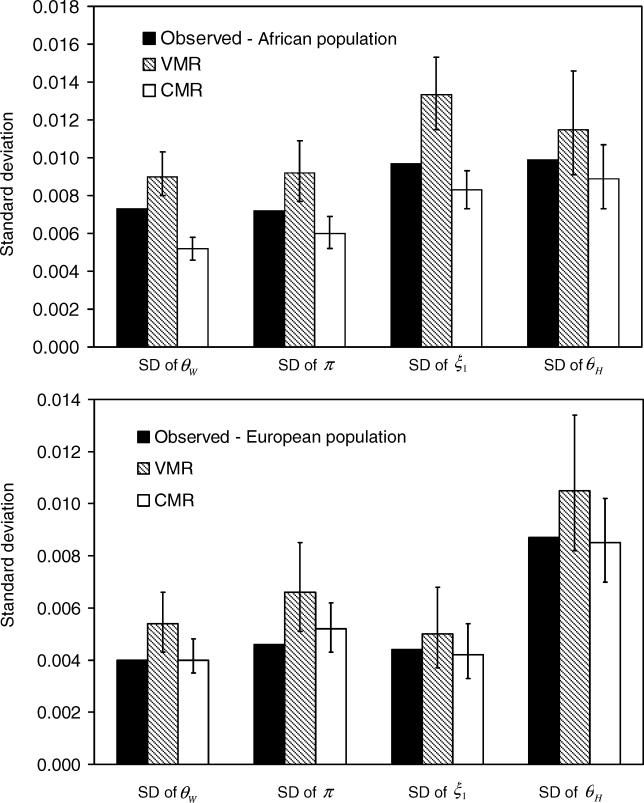
Evaluation of the Demographic Scenarios for the African and European Populations The standard deviation (SD) of a summary statistic is calculated as the standard deviation among loci. The values of the solid bars are obtained from the data. VMR, varying mutation rate model; CMR, constant mutation rate model. The 95% CI of the SD of each summary statistic obtained from 10^3^ simulated datasets is also given.

To further evaluate the estimated demographic scenarios of the two populations, we compared four summary statistics and their standard deviations calculated from the data to those expected under the demographic scenarios. The four summary statistics were chosen because they are components of three well-known neutrality tests [6–8]. The expected values of the summary statistics agree well with the observed ones in both scenarios (Table S1). The expected variances of all summary statistics among loci under the varying mutation rate model are larger than those under the constant mutation rate model, and the observed ones are between them in most cases (except for π in the European population) (Figure 4). Since the constant mutation rate model underestimates the variance of mutation rates among loci while the varying mutation rate model overestimates this variance, our estimated demographic scenarios account for most features of the standard deviations of the four summary statistics.

The time of the out-of-Africa migration we estimated in this study is probably too recent because gene exchange between Africa and Europe has been neglected in our model. On the other hand, the rate of gene flow was probably low because the estimated size of the founder population is very small. Furthermore, it is interesting to note that the out-of-Africa migration happened only about 44,000 y after the range expansion in Africa. This relatively long time suggests that the out-of-Africa migration may not be directly related to the range expansion in the ancestral African population.

### Inferring Selection: General Approach

The inferred demographic scenario is characterized by the best choice of parameter values (given the model) to explain the genome-wide polymorphism pattern in the data. However, we found that the MFSs expected under the demographic scenario still differ from the observed MFSs (Figure 3). The difference could be due to random effects and/or evolutionary forces acting locally in the genome (i.e., selection).

Since we observed an excess of rare and high-frequency derived mutations in both populations (relative to the inferred demographic scenarios), positive selection may play a role [6,8]. To reveal chromosomal regions affected by positive selection, we conducted a (nonoverlapping) window analysis using our LRT approach [12] with an important modification. Instead of employing the standard neutral model (of constant population size) as null hypothesis, we used the demographic scenarios estimated above. The alternative hypothesis is the hitchhiking model under the same demographic scenario. The hitchhiking model is parameterized by the variable position of the selected site (κ) and fixed values of 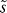 and 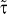 , where 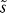 is the assigned selection coefficient and 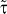 is the assigned time to the hitchhiking event in the past [12].

Besides inferring candidate regions affected by positive selection, it is of great interest to estimate the rate of adaptive substitution. However, due to false positives and relatively low power to detect weak and/or old selection events (Figures S1 and S2), the rate of adaptive substitution should not be estimated by counting the number of detected hitchhiking events. Thus, another approach is needed, as described in the following.

To obtain the rate of adaptive substitution and the distribution of selection coefficients in the African population, we summarized the polymorphism data of the *w*th window as **M** *_w_* (defined in Materials and Methods). We summarize each window according to the outcome of the LRTs because the outcome of the LRTs contains valuable information about the magnitude of *s* (Figures S1 and S2). Clearly, some information is lost using this approach, but it enables us to do the following analysis effectively.

Let δ be the rate of adaptive substitution, and the distribution of substitutions with selection coefficient *s* be denoted by *f(s)* where *s* > 0. It is assumed that δ is homogeneous over windows because all windows have the same length. We also assume that the windows are independent of one another. Then *L*(δ, *f(s)*) is given by 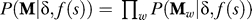 .

By dividing the outcomes for a window into neutral and selected cases, we have *P*(**M** *_w_*|δ, *f(s)*) = (1 − δ)*P*(**M** *_w_*|*neutral*) + δ∫*f(s)P*(**M** *_w_*|*s*)*ds* , where *P*(**M** *_w_*|*neutral*) and *P*(**M** *_w_*|*s*) are estimated by rejection sampling (described in Materials and Methods).

An obvious advantage of our approach is that we do not make any assumption about *f(s)*. Let 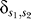 be defined as the rate of adaptive substitution with a selection coefficient within the interval (*s*_1_, *s*_2_). We have 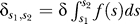 . Thus, δ and *f(s)* are estimated as {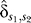 , 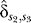 , …} and 
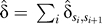
 , respectively, where *s*_1_ > 0, *s*_1_ < *s_i_*+1. Then the 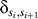 are estimated jointly using the likelihood function 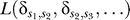 .

Furthermore, an LRT is used to test neutrality (i.e., whether 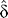 is significantly larger than 0). The null hypothesis is δ = 0 (there is no positive selection), and the alternative hypothesis is δ ≥ 0 (positive selection may exist).

### Identifying Candidate Regions of Positive Selection

We conducted a nonoverlapping window analysis to detect evidence for hitchhiking events (Tables S2 and S3). Each window has the same length (100 kb) and contains at least three loci. In total, 190 and 209 loci of the African and European samples, respectively, are considered. The varying mutation rate model is implemented in the LRT, and the published recombination rates [25] are used.

In the African sample, we observed that in 27.8% of the windows (15 of 54), the null hypothesis is rejected. Since the LRT may not be χ^2^ distributed, neutral simulated data are used to estimate the false-positive rate caused by the method. The false-positive rate (averaged over the windows) is 15.1%, and the multinomial test suggests that not all rejections of the null hypothesis are due to false positives (*p* < 0.05). In the European sample, the null hypothesis is rejected in 31.0% of the windows (18 of 58). The false-positive rate (averaged over the windows) is 21.0%, and the multinomial test also suggests that false positives cannot explain all rejections of the null hypothesis (*p* < 0.05).

In the African sample, 13 loci have a high mutation rate but relatively low diversity (Figure 2). The reduced diversity of these loci may be due to selective sweeps. Indeed, nine of the 13 loci are considered in the nonoverlapping window analysis (Table S4), and the null hypothesis (the neutral demographic expansion) is rejected in all windows in which these nine loci are located. Among these nine loci affected by positive selection and the four other fragments, there is only one locus with Tajima's *D* [6] and/or Fay and Wu's *H* [8] values that are significantly negative (Table S4). It suggests that our proposed test [12] has higher power to detect selective sweeps than Tajima's *D* [6] and Fay and Wu's *H* [8].

For the European population, we compared the windows in which the null hypothesis is rejected (Table S3) with the outlier approach by Ometto et al. [18]. They found eight outliers, which cannot be explained by demography alone. Seven of them are included in our window analysis, and the neutral null hypothesis is rejected in all windows in which these seven outliers are located.

We also tested the null hypothesis (δ = 0) against the alternative hypothesis (δ ≥ 0). The LRT suggests that the demographic scenario alone cannot account for the polymorphism feature of both the African and European populations because the hypothesis of δ = 0 is rejected in both populations (*p* < 0.05, *df* = 1, χ^2^_0.05_ = 3.84).

### Rate of Adaptive Substitution and the Distribution of Selection Coefficients

**Figure 5:**
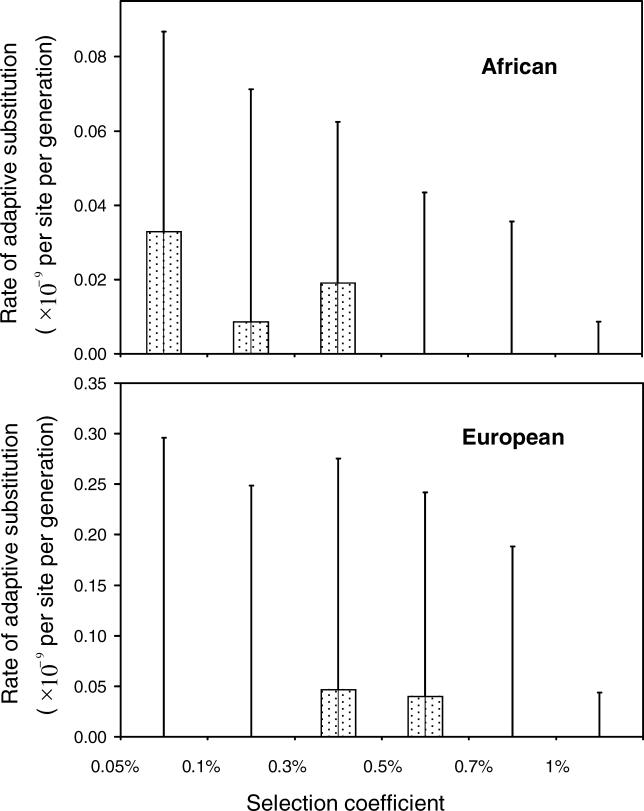
The Frequency Spectrum of Selection Coefficients *(s)* for Recent Positive Selection in the African and European Populations The *y*-axis shows the rate of adaptive substitution with an *s* value in (*s_1_*, *s_2_*) if all selectively driven substitutions occur in coding regions. The 95% likelihood-based CIs are given as error bars.

Following the methods outlined above and more fully described in Materials and Methods, the frequency spectra of selection coefficients for both populations were obtained (Figure 5). The frequency spectra are incomplete since we cannot infer sweeps with *s* < 0.05% due to a lack of power to detect them (Figures S1 and S2). The spectra suggest that larger *s* values are less frequent. Few *s* values are larger than 0.5% and 0.7% in the African and European populations, respectively. These upper bounds cannot be attributed to the method, as the full LRT has reasonable power to detect selective sweeps when selection is strong (Figures S1 and S2). Furthermore, the estimated selection coefficients of the African population are smaller than those in the European population. Given that the effective population size of the African population is about 8-fold larger than that of the European one (which leads a larger value of α = 2*Ns*), this result is not surprising.

In the African population, the estimated rate of adaptive substitution (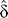 ) is 0.0117 × 10^−9^ per site per generation with a 95% CI of (0.0025 × 10^−9^, 0.0167 × 10^−9^). Here (and also for the European population, see below), the upper bound of the CI is determined by the assumption that at most one hitchhiking event occurs within a window (100 kb). Furthermore, since the CIs obtained do not include the uncertainty in the inferred demographic scenarios, they may be too narrow. We extrapolated these results to the entire euchromatic portion of the X chromosome of D. melanogaster (the length of which is 22.22 Mb, FlyBase, Release 4.2, http://www.flybase.org). Thus, we estimated that about 160 beneficial mutations have fixed during the last 60,000 y (i.e., since population size expansion). To compare our results with a previous estimate that has been inferred from data on coding regions [4], we assume that all selectively driven substitutions occur in coding regions. Since these comprise about 19.2% of the windows analyzed, the rate of adaptive substitution is 0.061 × 10^−9^ (0.013 × 10^−9^, 0.087 × 10^−9^) per site per generation. The size of the CI is mainly determined by the number of beneficial mutations that have been fixed during the last 60,000 y within the windows considered.

In the European population, the estimated rate of adaptive substitution is 0.0168 × 10^−9^ (0.0026 × 10^−9^, 0.0646 × 10^−9^) per site per generation. Thus, we estimated that about 60 beneficial mutations fixed on the X chromosome in the derived European population. If these substitutions occurred exclusively in coding regions, the rate of adaptive substitution is 0.088 × 10^−9^ (0.014 × 10^−9^, 0.336 × 10^−9^) per site per generation. Since the number of beneficial substitutions in the derived European population is smaller than that in the African population, it is reasonable that the CI of 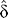 in the European population is wider than that in the African population.

Our analysis suggests that positive selection may have been important for the African population in the past 60,000 y since it expanded its size. This is not surprising as during that time period dramatic environmental changes occurred in Africa with a transition from a full glacial to an interglacial period (70 to 55 kya [26]) and a last glacial maximum in the late Pleistocene (18 to 21 kya [27]). Signatures of positive selection in the African population have also been reported elsewhere [28].

Because the European population is derived very recently, it is reasonable that we observed a smaller number of beneficial substitutions than in the African population, although the estimated rate of adaptive substitution appears to be higher in Europe. Our analysis also suggests that the genome-wide reduction of variability in the European population (relative to the African one, Table S1) is mainly due to bottlenecks rather than selection.

It is likely that the estimated rate of adaptive substitution does not depend on the lengths and positions of the windows since similar estimates for the African population were obtained when longer windows of 120 and 150 kb were used. Furthermore, since only few beneficial mutations with strong selection coefficients exist (Figure 5), we suggest that recent strong selection (affecting genetic polymorphism within multiple windows) does not affect the estimated rate of adaptive substitution. We confirmed this by checking the pattern of polymorphism in the African population along an 11-Mb region with densely spaced loci (from Fin114 to Fin432; see [16,18]). We did not observe a “valley” of reduced polymorphism spanning multiple windows (results not shown).

The frequent occurrence of weak (perhaps undetected) sweeps in the African population may have an impact on the estimated demographic expansion scenario because they contribute to the genome-wide skew of the MFS. A large number of undetectable sweeps was also found using other methods [29]. However, this would make our method for detecting selection more conservative.

Our inference of positive selection could also be biased because of the presence of purifying selection [28]. When deleterious mutations occur, the probability of fixation of beneficial mutations will be reduced. Furthermore, since we ignored the interaction among beneficial mutations, the efficacy of selection may be reduced. Thus, the frequency distributions of selection coefficients may be shifted to smaller values.

There are several other reasons why the rates of adaptive substitution could be underestimated. (a) Using our approach, we cannot infer weak selection events (*s* < 0.05%). (b) We only considered adaptation from new mutations [30–33] in our study. However, adaptation from standing genetic variation [34,35] may have also played a substantial role in the colonization of new habitats (in particular, Europe). Since the reduction of polymorphism under adaptation from standing genetic variation is generally smaller than that under new mutations [35], multiple adaptations from standing genetic variation may have a similar effect as an adaptation from a single new mutation. (c) We assumed that there is only one adaptation from a new mutation within a window, which may be invalid. If so, the rate of adaptive substitution may be underestimated, and the distribution of selection coefficients may be biased toward larger values. (d) We considered only one demographic scenario per population. The MFS under other demographic scenarios may depart more from the observed MFS (Figures 3 and S3). Thus, more selection events may be needed to explain the larger discrepancies.

Despite these caveats, our estimated rates of strong adaptive substitutions are only slightly lower than the published rate (0.092 × 10^−9^ per site per generation [4]), which was obtained by averaging over a long time period (since the divergence of D. melanogaster and D. simulans). Considering the fact that the published rate [4] also takes relatively weak selection into account, our results may indicate an accelerated rate of adaptation in both populations due to recent environmental changes.

## Materials and Methods

### DNA polymorphism data.

We analyzed noncoding DNA polymorphism on the X chromosome from two regional D. melanogaster populations: the Netherlands and Zimbabwe [16–18]. The positions of the loci and the inference of coding regions are based on FlyBase, Release 4.2 (http://www.flybase.org). Two loci (Fin450 and Fin311) are partly overlapping according to the latest annotation, so that Fin311 is discarded due to its shorter length. Therefore, the number of loci is 272 and 262 in the European and African samples, respectively. Among the loci of the European sample, 113 loci belong to intergenic regions, 158 to introns, and one to an untranslated transcribed region (UTR). Of the loci of the African sample, 110 loci belong to intergenic regions, 151 to introns, and one to an UTR. The sample sizes of the datasets by Glinka et al. [16] and Ometto et al. [18] are nine to 12 for both populations, and those of Haddrill et al. [17] (ten loci) are 16 to 23. The average sequence length is 510 bp. The sequences used in this study were obtained from the EMBL Nucleotide Sequence Database and GenBank. The program Recomb-Rate [25] was used to obtain the local recombination rate. The homologous sequences of D. simulans are used as outgroup data, and insertion/deletions are excluded from the analysis.

The divergence between D. melanogaster and D. simulans is calculated by Kimura's [36] method, where evolutionary rates among sites are modeled using the Gamma distribution [37]. The averages of the summary statistics θ*_W_*, π, ξ_1_, and θ*_H_* are calculated according to [38] because of different sample sizes and sequence lengths of the loci, where θ*_W_* is Watterson's estimator of θ, π is the nucleotide diversity, ξ_1_ is the number of singletons (derived mutations that are carried by one sampled chromosome), and θ*_H_* is the summary statistics in Fay and Wu's *H* test [8].

### Demographic model.

We assume that the demographic history of the Zimbabwe population is characterized by an expansion model (Figure 1). That is, the population size was constant, but there was an instantaneous increase of effective population size from *N_A1_* to *N_A0_* at time *t_A0_* ago, where the time is scaled such that one unit represents 2*N_A0_* generations. Thus, the expansion model is characterized by 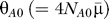 , *t_A0_*, and the strength of the expansion (*N_A_*_0_/*N_A_*_1_).

For inferring the demographic history of the European population, a two-population model is used (Figure 1). The African and European populations diverged at time *t_E0_* + *t_E1_* ago (scaled by 2*N_E0_* generations). One population of size *N_E1_* moved out of Africa. We assume that the size of the founder population is small compared with the size of the African population, so that the size of the African population remains unchanged. After time *t_E1_*, the size of the founder population expanded to *N_E0_* instantaneously. Thus, the bottleneck model in the European population is characterized by 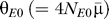 , *t_E0_*, *t_E1_*, and the strength of the bottleneck (*N_E_*_0_/*N_E_*_1_).

### Coalescent simulation.

It is assumed that there is no recombination within a locus; thus each locus is treated as a point in the ancestral recombination graph (ARG). An ARG for the partly linked loci within the considered DNA region is constructed by coalescence [39–41]. The recombination rate is given by [25]. Only a minor revision is needed to implement the proposed demographic models. Selection is modeled as follows. Denoting *b* as the wild-type allele and *B* as the favored allele at the selected locus, the three genotypes, *bb, bB,* and *BB,* have the relative fitnesses 1, 1 + *s,* and 1 +2*s,* respectively.

The proposed methods for constructing an ARG under the Wright-Fisher model [39–41] assume that the recombination and coalescent events do not occur within one generation. This assumption may be invalid when the neutral loci are spread across a very large genomic region or the whole chromosome. Therefore, for all loci on the X chromosome, we construct the ARG under the Wright-Fisher model generation by generation. The coalescence probability of two lineages within one generation is given by [39]. Since multiple coalescent events could happen within one generation, the coalescence process is implemented by random sampling. The recombination process follows the Poisson distribution.

The coalescence under the hitchhiking model is divided into three phases: a neutral phase, a selective phase, and a second neutral phase. To include the proposed demographic models, the treatment of the selective phase follows previous work [9,42] with minor revision. It is assumed that the frequency of the beneficial allele increases from λ to (1 − λ) in a population of varying size, where λ = 1/2*N*_0_, and *N*_0_ is the current effective population size for the X chromosome. The neutral phases are modeled as described above.

A chromosomal region in the European sample could be affected by two hitchhiking events: a recent one happened in the European population after the two populations split, and another independent one occurred in the ancestral African population before the split. To simulate this case, we divide the coalescent into five phases accordingly: a neutral phase, a recent selective phase in the European population, a second neutral phase, a selective phase in the ancestral African population, and a third neutral phase.

### LRT and likelihood-based CIs.

The LRT is a statistical test of the goodness-of-fit between two models. If the null model (the simple model) and the alternative model (the complex model) are hierarchically nested, and the null model has one parameter less than the alternative model, then we have χ^2^ = −2ln(max *L_null_*/max *L_alternative_*), and this LRT statistic may approximately follow a χ^2^ distribution with 1 *df.*

In case of a multiparameter model (**ϑ**_1_, **ϑ**_2_, …, **ϑ** *_k_*), we may be interested in the CI of one parameter at a time, say in ϑ_1_. Let 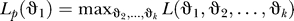 be the profile likelihood. Then the likelihood-based approximative 95% CI is 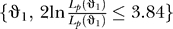 , where 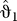 is the maximum likelihood estimate of **ϑ**_1_ [43].

Similarly, when we are interested in the CI of **ϑ** *_sum_* (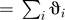 ), we have 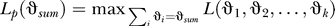 . Then the likelihood-based approximative 95% CI is 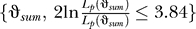 , where 
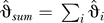
 [43].

### Maximum likelihood method for inferring the demographic scenarios.

The MFS of 262 loci represents the data for inferring the demographic scenario of the African population, and the joint MFS of 272 loci the data for inferring the demographic scenario of the European population.

When analyzing the African sample, the genealogies are simulated conditional on the joint parameters of the demographic expansion model. The method can be easily modified when the outgroup sequence is not available. In such a case, we cannot distinguish mutations carried by *i* sampled chromosomes from those carried by (*n* – *i*) sampled chromosomes, and thus 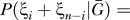 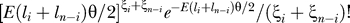 .

The likelihood of the joint MFS is used to infer the demographic scenario of the European population given the demographic scenario of the African population. The likelihood for the *k*th locus is 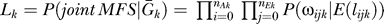 , where *nAk* and *n_Ek_* are the sample sizes of the *k*th locus for the African and European populations, respectively, and ω*_ijk_* is the number of derived mutations carried by *i* sampled chromosomes in the African sample and by *j* sampled chromosomes in the European sample at the *k*th locus. Furthermore, we have 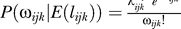 , where *E(l_ijk_)* is the expected length of branches with *i* African descendants and *j* European descendants at the *k*th locus under the demographic scenario, and λ*_ijk_* = *E(l_ijk_)*θ*_Ek_*/2, and θ*_Ek_* = 4*N_E_*_0_μ*_k_*. Here, the branch length is scaled so that one unit represents 2*N_E_*_0_ generations. Since loci are independent given the expected branch lengths, the likelihood for all loci is 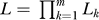 , where *m* is the number of loci.

In this study, the expected branch length is obtained through Monte-Carlo simulations. Obviously, the accuracy of the estimation can be improved using large numbers of simulations *(K).* We ran *K* = 10^6^ simulations for inferring the demographic scenarios of the African and European populations.

The likelihood-based CI of a parameter is obtained by the method described above. We evaluated the validity of the likelihood-based CI using simulated data. By comparing the likelihood-based CI with the CI estimated from simulated data, we found that the difference between both CIs is reasonably small (results not shown).

### Identifying candidate regions of positive selection.

The loci within the same window are partly linked, and the recombination rate between loci is given by [25]. The windows are listed in Tables S2 and S3. The compact MFS is used to detect positive selection after a revision of the proposed likelihood method *(L1)* [12]. Instead of employing the standard neutral model (of constant population size) as null hypothesis, we used the demographic scenario estimated above. The alternative hypothesis is the hitchhiking model under the same demographic scenario.

We denote 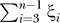 as ξ*_X_*, where ξ*_i_* is the number of derived mutations carried by *i* sampled chromosomes. Then, the compact MFS over *m* partly linked loci [12] is defined as

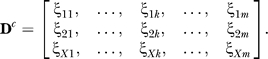



Following Felsenstein and his colleagues [44,45], the probability that **D** *^c^* is observed given the position of the selected site (κ) is

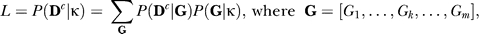
and *G_k_* is the genealogy for the *k*th locus conditioned on κ, 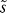 , 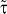 , the recombination rates among loci [25], and the demographic scenario.

To obtain the likelihood, it requires a summation over a huge number of topologies, and each topology has an infinite number of possible branch lengths. Therefore, rather than sampling all genealogies, we consider a large random sample of **G**. The approach is possible and efficient because each simulated **G** is consistent with **D** *^c^* when *n* ≥ 5 [12]. Since *P*(**G**|κ) is determined in the simulation process, an estimate of *L* can be obtained by the following procedure:

1. Simulate genealogies (topology without mutation) for *m* loci conditioned on κ, 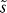 , 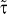 , the recombination rates among loci, and the demographic scenario.
2. Compute the value of *L*_G_ as

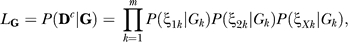
where *P*(ξ*_i_*|*G*) is given by the Poisson probability [12].
3. Repeat steps 1 and 2 *K* times. Then 
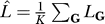
 . Here, we use *K* = 10^5^.

In this study, we used six hitchhiking models that differ in 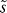 (= 0.05, 0.1, 0.2, 0.5, 1, 5; all values in percent), and we put 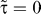 . If one or more hitchhiking models are accepted by the LRTs in a window, the window is considered a candidate for a selective sweep. Since the LRT can be affected by a “sweep-like” polymorphism pattern observed under a neutral demographic scenario, false-positive cases can occur. For each window, the false-positive rate is estimated from 10^3^ simulated neutral data sets (conditioned on the recombination rates among loci and the demographic scenario). It is the probability that one or more hitchhiking models are accepted by the LRTs (based on simulated neutral data).

The multinomial test is used to test whether all candidates of selective sweeps are due to false positives. This is given by the probability that the number of false positives in the windows is larger than or equal to the number of candidates.

Since simulations suggest that the variance of the summary statistics θ*_W_*, π, ξ_1_, and θ*_H_* among the loci of the X chromosome is not sensitive to the current effective population size, we set the current effective population size as 100,000 to simulate genealogies effectively when we conduct the LRTs.

### Estimating δ and *f(s)* for the African population.

Let **M** *_w_* = [*w*_1_,…,*w*_6_] comprise the summary statistics of the LRTs (at the 5% significant level) for the *w*th window, where *w_i_* denotes the result of the LRT when the *i*th hitchhiking model (i.e., that with the *i*th 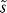 value, sorted from minimum to maximum) is used. *w_i_* is 1 if the null hypothesis is rejected and 0 if not. For all of the analyzed windows, the matrix of summary statistics of the LRTs is

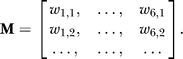



Thus, 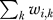 is the number of windows in which the null hypothesis is rejected in the sample when the *i*th hitchhiking model is used.

We assume that the windows are independent of one another. Then 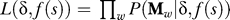 , where *s* should not be confused with the fixed value of 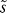 . By dividing the outcomes for a window into neutral and selected cases, we have *P*(**M** *_w_*|δ, *f(s)*) = (1 − δ)*Q(neutral)* + δ∫*f(s)Q(s)ds*, where *Q(neutral) = P*(**M** *_w_*|*neutral*) and *Q(s)* = *P*(**M** *_w_*|*s*). Deriving a general formula for *Q* appears to be analytically intractable, but an estimate can be obtained from the following rejection sampling procedure:

Step 1. The polymorphism data of loci within a window are simulated conditional on the demographic scenario, the recombination rates among loci [25], and 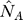 . Under the hitchhiking case, the simulation is also conditional on κ, *s*, and τ. κ is randomly distributed across coding regions within the window, and τ is uniformly distributed within [0, *t_A0_*]. Since *t_A0_* is estimated to be close to the limit of detection (which is approximately 0.1*N_e_* generations [9,14]), older hitchhiking events are not expected to have contributed much to the patterns in the data. The simulated data are then analyzed by the LRTs, and the results are summarized in [*w*_1,*sim*_,…,*w*_6,*sim*_].

Step 2. We introduce the indicator variable

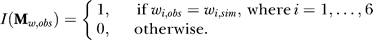



Step 3. Repeat the steps 1 and 2 *B* times. The probability 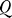 is then approximated by 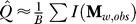 . We use *B* = 1,000 in this study.

Therefore, *Q(neutral)* > *Q(s)* is expected if the data are simulated under neutrality. If the hitchhiking data with selection coefficient *s*′ are simulated, we expect that a maximum *Q(s)* (where *s* > 0) could be obtained when *s* = *s*′. In this study, *Q(s)* is calculated for 18 values of *s* (i.e., 0.06, 0.08, 0.09, 0.1, 0.2, 0.3, 0.4, 0.5, 0.6, 0.7, 0.8, 0.9, 1, 2, 4, 6, 8, 10; all values in percent).

Then we partition the value of *s* (given in percentage) into six regions, which are [0.05, 0.1), [0.1, 0.3), [0.3, 0.5), [0.5, 0.7), [0.7, 1), and [1, 20). Thus, to estimate δ and *f(s),* we need to estimate six parameters (δ_0.05,0.1_, δ_0.1,0.3_, δ_0.3,0.5_, δ_0.5,0.7_, δ_0.7,1_, and δ_1,20_), and δ is given by their summation. We treat the cases (*s* < 0.05%) as neutral because of the very low power to detect them (Figures S1 and S2). To maximize the likelihood, likelihoods in a six-dimensional parameter space are calculated, where each dimension represents one parameter. To be specific, the minimum and maximum values of a parameter are 0 and 0.0167 × 10^−9^ per site per generation (see above), respectively. The spacing of the grid of parameter values is 0.0003 × 10^−9^ per site per generation.

### Estimating δ and *f(s)* for the European population.

The genetic polymorphism in the European sample could be affected by sweeps that occurred in the ancestral African population before the split. Thus, we need to consider the effect of “old” sweeps when estimating δ*_E_* and *f(s_E_)*. Here, we use the indices of *A* and *E* to distinguish the parameters for the European and the African populations.

For hitchhiking events that occurred in the derived European population, τ is uniformly distributed within [0, *t_E0_*]. To estimate δ*_E_* and *f(s_E_),* we divide the outcomes for a window in the European sample into four cases: (a) there is no sweep; (b) a sweep occurred in the European population after the split; (c) a sweep occurred in the ancestral African population before the split; and (d) a sweep occurred in the European population after the split, and another sweep in the ancestral African population before the split.

Given a sweep originated in the African population, the probability that the sweep occurred before the split is η = (*t_A_*_0_ − *t_E_*_0_ − *t_E_*_1_)/*t_A_*_0_. Then, the probability 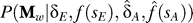 is given by

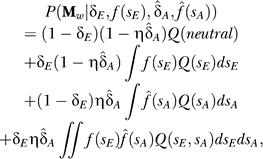
where 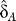 and 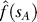 are known parameters estimated from the African sample, and *Q*(*s_E_, s_A_*) = *P*(**M** *_w_*|*s_E_, s_A_*).

The related *Q* is estimated by the method described above. When we estimate *Q*(*s_E_, s_A_*), we use *B* = 100.

## Supporting Information

Figure S1The Power of the LRT to Detect Sweeps in the African SampleThe length of each window is 100 kb, and the power is obtained by averaging over the windows. *s* is the true value under which the data are simulated, and 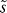 is the assigned (fixed) value in the hitchhiking model. The values of *s* and 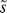 are given in percentage.
(168 KB DOC)

Figure S2 The Power of the LRT to Detect Sweeps in the European Population (164 KB DOC)

Figure S3The Comparison of Derived MFS under Different Population Expansion Scenarios in the African PopulationMaximum likelihood estimates: 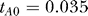 , and the strength of the expansion = 5.0. The other three expansion scenarios are chosen such that the parameter values are within the estimated CIs. Expansion1: 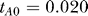 , and the strength of the expansion = 4.0; Expansion2: 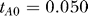 , and the strength of the expansion = 6.0; Expansion3: 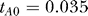 , and the strength of the expansion = 8.0. The sum of the squares of the residuals (between the expected and the observed) is 0.0012, 0.0066, 0.0025 and 0.0035, respectively.
(81 KB DOC)

Table S1 Evaluation of Demographic Models for the African and European Populations (26 KB DOC)

Table S2 Results of the Nonoverlapping Window Analysis of the African Sample Based on Different Hitchhiking Models (124 KB DOC)

Table S3 Results of the Nonoverlapping Window Analysis of the European Sample Based on Different Hitchhiking Models (122 KB DOC)

Table S4 List of 13 Loci Which Have High Mutation Rate but Low Diversity in the African Sample (44 KB DOC)

### Accession Numbers

The sequences used in this study were obtained from the EMBL Nucleotide Sequence Database (http://www.ebi.ac.uk/embl) (AJ568984 to AJ571588, AJ568984 to AJ571588) and GenBank (http://www.ncbi.nlm.nih.gov/Genbank) (AY925214 to AY926258).

## Abbreviations

ARG: ancestral recombination graph
CI: confidence interval
kya: 1,000 years ago
LRT: likelihood ratio test
MFS: mutation frequency spectrum
