# Supplementary material for: Inferring the Demographic History and Rate of Adaptive Substitution in Drosophila
Source: PLoS Genet. 2006 Oct 13;2(10):e166. doi: 10.1371/journal.pgen.0020166 (PMC1599771; doi:10.1371/journal.pgen.0020166)
Supplement: Supplementary file 1 [file pgen.0020166.sg001.doc]

Figure S1. The power of the LRT to detect sweeps in the African sample. The length of each window is 100 kb, and the power is obtained by averaging over the windows. *s* is the true value under which the data are simulated, and $\tilde{s}$ is the assigned (fixed) value in the hitchhiking model. The values of *s* and $\tilde{s}$ are given in percentage.
