# Supplementary material for: Inferring the Demographic History and Rate of Adaptive Substitution in Drosophila
Source: PLoS Genet. 2006 Oct 13;2(10):e166. doi: 10.1371/journal.pgen.0020166 (PMC1599771; doi:10.1371/journal.pgen.0020166)
Supplement: Supplementary file 2 [file pgen.0020166.sg002.doc]

Figure S2. The power of the LRT to detect sweeps in the European population.
