# Supplementary material for: Inferring the Demographic History and Rate of Adaptive Substitution in Drosophila
Source: PLoS Genet. 2006 Oct 13;2(10):e166. doi: 10.1371/journal.pgen.0020166 (PMC1599771; doi:10.1371/journal.pgen.0020166)
Supplement: Supplementary file 3 [file pgen.0020166.sg003.doc]

Figure S3. The comparison of derived mutation frequency spectrum (MFS) under different population expansion scenarios in the African population. Maximum likelihood estimates: $t_{A0}=0.035$, and the strength of the expansion = 5.0. The other three expansion scenarios are chosen such that the parameter values are within the estimated confidence intervals. Expansion1: $t_{A0}=0.020$, and the strength of the expansion = 4.0; Expansion2: $t_{A0}=0.050$, and the strength of the expansion = 6.0; Expansion3: $t_{A0}=0.035$, and the strength of the expansion = 8.0. The sum of the squares of the residuals (between the expected and the observed) is 0.0012, 0.0066, 0.0025 and 0.0035, respectively.
