# Supplementary material for: Inferring the Demographic History and Rate of Adaptive Substitution in Drosophila
Source: PLoS Genet. 2006 Oct 13;2(10):e166. doi: 10.1371/journal.pgen.0020166 (PMC1599771; doi:10.1371/journal.pgen.0020166)
Supplement: Supplementary file 4 [file pgen.0020166.st001.doc]

Table S1. Evaluation of demographic models for the African and European populations.

|  | $\bar{\theta}_{W}$ | $\bar{\pi}$ | $\bar{\xi}_{1}$ | $\bar{\theta}_{H}$ |
| --- | --- | --- | --- | --- |
| African population | | | | |
| Observed^a^ | 0.0126 | 0.0110 | 0.0172 | 0.0118 |
| EP-VMR^b^ | 0.0126 | 0.0114 | 0.0171 | 0.0101 |
| EP-CMR^b^ | 0.0125 | 0.0114 | 0.0170 | 0.0101 |
| European population | | | | |
| Observed^a^ | 0.0046 | 0.0047 | 0.0036 | 0.0074 |
| BN-VMR^b^ | 0.0047 | 0.0051 | 0.0033 | 0.0073 |
| BN-CMR^b^ | 0.0046 | 0.0050 | 0.0033 | 0.0072 |

^a^ The values are obtained from the data.

^b^ The expansion model (EP) represents the estimated demographic scenario of the African population. The bottleneck model (BN) represents the estimated demographic scenario of the European population. VMR: varying mutation rate model; CMR: constant mutation rate model.
