# Supplementary material for: Inferring the Demographic History and Rate of Adaptive Substitution in Drosophila
Source: PLoS Genet. 2006 Oct 13;2(10):e166. doi: 10.1371/journal.pgen.0020166 (PMC1599771; doi:10.1371/journal.pgen.0020166)
Supplement: Supplementary file 5 [file pgen.0020166.st002.doc]

Table S2. Results of the non-overlapping window analysis of the African sample based on different hitchhiking models^a^.

| Window Index | Start Position | Hitchhiking models ($\tilde{s}$) | | | | | |
| --- | --- | --- | --- | --- | --- | --- | --- |
|  |  | 0.05% | 0.1% | 0.2% | 0.5% | 1% | 5% |
| 1 | 1,963,914 | 0 | 0 | 1 | 0 | 1 | 1 |
| 2 | 2,217,745 | 0 | 0 | 0 | 0 | 0 | 0 |
| 3 | 3,337,374 | 0 | 0 | 0 | 0 | 0 | 0 |
| 4 | 3,623,869 | 0 | 0 | 0 | 0 | 0 | 0 |
| 5 | 3,865,135 | 0 | 0 | 0 | 0 | 0 | 0 |
| 6 | 5,072,787 | 0 | 0 | 0 | 0 | 0 | 0 |
| 7 | 6,596,448 | 0 | 0 | 1 | 1 | 1 | 0 |
| 8 | 6,739,669 | 0 | 0 | 0 | 0 | 0 | 0 |
| 9 | 6,995,330 | 0 | 0 | 0 | 0 | 0 | 0 |
| 10 | 7,717,199 | 0 | 0 | 0 | 0 | 0 | 0 |
| 11 | 9,142,259 | 0 | 0 | 0 | 1 | 0 | 0 |
| 12 | 9,844,084 | 0 | 0 | 0 | 0 | 0 | 0 |
| 13 | 10,080,656 | 0 | 0 | 0 | 0 | 0 | 0 |
| 14 | 10,248,469 | 0 | 1 | 1 | 1 | 1 | 1 |
| 15 | 10,374,457 | 0 | 1 | 1 | 1 | 1 | 1 |
| 16 | 10,487,961 | 0 | 0 | 0 | 0 | 0 | 0 |
| 17 | 10,588,418 | 0 | 0 | 0 | 0 | 0 | 0 |
| 18 | 10,722,208 | 0 | 1 | 1 | 1 | 0 | 0 |
| 19 | 10,871,654 | 0 | 0 | 0 | 0 | 0 | 0 |
| 20 | 11,011,357 | 0 | 0 | 0 | 0 | 0 | 0 |
| 21 | 11,124,111 | 0 | 0 | 0 | 0 | 0 | 0 |
| 22 | 11,234,744 | 0 | 0 | 0 | 0 | 0 | 0 |
| 23 | 11,411,043 | 0 | 0 | 0 | 0 | 0 | 0 |
| 24 | 11,531,088 | 0 | 0 | 0 | 1 | 0 | 0 |
| 25 | 11,623,558 | 0 | 0 | 0 | 0 | 0 | 0 |
| 26 | 11,884,635 | 0 | 0 | 0 | 0 | 0 | 0 |
| 27 | 12,012,791 | 0 | 0 | 0 | 0 | 0 | 0 |
| 28 | 12,139,521 | 0 | 0 | 0 | 0 | 0 | 0 |
| 29 | 12,553,962 | 0 | 0 | 0 | 0 | 0 | 0 |
| 30 | 12,689,474 | 0 | 0 | 0 | 0 | 0 | 0 |
| 31 | 12,857,509 | 0 | 0 | 1 | 1 | 1 | 1 |
| 32 | 13,004,660 | 0 | 0 | 0 | 0 | 0 | 0 |
| 33 | 13,225,703 | 0 | 0 | 0 | 0 | 0 | 0 |
| 34 | 13,339,925 | 0 | 0 | 0 | 0 | 0 | 0 |
| 35 | 13,645,268 | 0 | 0 | 0 | 0 | 0 | 0 |
| 36 | 14,124,430 | 0 | 0 | 1 | 0 | 0 | 0 |
| 37 | 14,298,012 | 0 | 0 | 0 | 0 | 0 | 0 |
| 38 | 14,414,901 | 0 | 0 | 0 | 0 | 0 | 0 |
| 39 | 14,509,968 | 0 | 0 | 0 | 0 | 0 | 0 |
| 40 | 14,614,782 | 0 | 0 | 0 | 0 | 0 | 0 |
| 41 | 14,912,857 | 0 | 0 | 0 | 0 | 0 | 0 |
| 42 | 15,054,940 | 0 | 1 | 1 | 0 | 0 | 0 |
| 43 | 15,456,035 | 0 | 0 | 0 | 1 | 1 | 0 |
| 44 | 15,596,331 | 0 | 1 | 1 | 0 | 0 | 0 |
| 45 | 15,714,571 | 0 | 0 | 0 | 0 | 0 | 0 |
| 46 | 15,901,121 | 0 | 0 | 0 | 0 | 0 | 0 |
| 47 | 16,082,545 | 0 | 0 | 0 | 1 | 0 | 0 |
| 48 | 16,252,614 | 0 | 0 | 0 | 0 | 0 | 0 |
| 49 | 16,369,947 | 0 | 0 | 0 | 0 | 0 | 0 |
| 50 | 16,460,891 | 0 | 0 | 0 | 0 | 0 | 0 |
| 51 | 16,849,070 | 0 | 0 | 0 | 0 | 0 | 0 |
| 52 | 17,007,895 | 0 | 1 | 1 | 0 | 0 | 0 |
| 53 | 17,292,864 | 0 | 0 | 0 | 0 | 0 | 0 |
| 54 | 17,441,743 | 0 | 0 | 0 | 1 | 0 | 0 |

^a^ The length of each window is 100 kb. 1 means that the hitchhiking model is favored by the likelihood ratio test. 0 means that the null hypothesis is accepted by the likelihood ratio test.
