# Supplementary material for: Inferring the Demographic History and Rate of Adaptive Substitution in Drosophila
Source: PLoS Genet. 2006 Oct 13;2(10):e166. doi: 10.1371/journal.pgen.0020166 (PMC1599771; doi:10.1371/journal.pgen.0020166)
Supplement: Supplementary file 7 [file pgen.0020166.st004.doc]

Table S4. List of 13 loci which have high mutation rate but low diversity in the African sample.

| ID of Locus | Position | Length | *n* | Tajima’s *D* | Fay & Wu’s *H* | Window Index |
| --- | --- | --- | --- | --- | --- | --- |
| Fin018 | 2,555,495 | 487 | 12 | -0.501 | -3.727 |  |
| Fin073 | 5,592,462 | 562 | 12 | -0.872 | 1.758 |  |
| Fin114 | 6,605,700 | 285 | 12 | -0.379 | -0.424 | 7 |
| Fin115 | 6,651,455 | 366 | 12 | -0.964 | 1.152 | 7 |
| Fin165 | 9,202,487 | 251 | 12 | -0.781 | -1.576 | 11 |
| Fin169 | 9,420,312 | 279 | 12 | -0.850 | 0.394 |  |
| Fin188 | 10,324,198 | 488 | 12 | -1.385 | 0.697 | 14 |
| Fin191 | 10,439,282 | 432 | 12 | 0.385 | -0.152 | 15 |
| Fin475 | 10,746,846 | 390 | 12 | -1.129 | 0.909 | 18 |
| Fin389 | 15,057,271 | 501 | 12 | -1.001 | 1.606 | 42 |
| Fin393 | 15,169,667 | 555 | 12 | -0.474 | 0.091 |  |
| Fin295 | 15,544,489 | 424 | 12 | -1.983* | -6.061* | 43 |
| Fin296 | 15,599,496 | 578 | 12 | -0.840 | 0.727 | 44 |

* Tajima’s *D* or Fay & Wu’s *H* is significantly negative or positive (*p* < 0.05, one-tailed).
